# Fatal Elephant Endotheliotropic Herpesvirus Infection of Two Young Asian Elephants

**DOI:** 10.3390/microorganisms7100396

**Published:** 2019-09-26

**Authors:** Selvaraj Pavulraj, Kathrin Eschke, Adriane Prahl, Michael Flügger, Jakob Trimpert, Petra B. van den Doel, Sandro Andreotti, Sabine Kaessmeyer, Nikolaus Osterrieder, Walid Azab

**Affiliations:** 1 Institut für Virologie, Robert von Ostertag-Haus, Zentrum für Infektionsmedizin, Freie Universität Berlin, Robert-von-Ostertag-Str. 7–13, 14163 Berlin, Germany; pavulraj1vet@zedat.fu-berlin.de (S.P.); Kathrin.Eschke@fu-berlin.de (K.E.); Jakob.Trimpert@fu-berlin.de (J.T.); no.34@fu-berlin.de (N.O.); 2 Tierpark Hagenbeck gem. GmbH, Lokstedter Grenzstraße 2, 22527 Hamburg, Germany; Adriane.Prahl@hagenbeck.de (A.P.); Michael.Fluegger@hagenbeck.de (M.F.); 3 ViroScience Lab, Erasmus Medical Center, Erasmus MC, Room Ee1714, dr. Molewaterplein 50, Rotterdam, 3015, GE, The Netherlands; p.vandendoel@erasmusmc.nl; 4 Department of Mathematics and Computer Science, Institute of Computer Science, Freie Universität Berlin, Arnimallee 14, 14195 Berlin, Germany; Sandro.Andreotti@fu-berlin.de; 5 Department of Veterinary Medicine, Institute of Veterinary Anatomy, Freie Universität Berlin, Koserstraße 20, 14195 Berlin, Germany; Sabine.Kaessmeyer@fu-berlin.de

**Keywords:** EEHV-1A, herpesvirus, elephant, cell culture, diagnosis, gB

## Abstract

Elephant endotheliotropic herpesvirus (EEHV) can cause a devastating haemorrhagic disease in young Asian elephants worldwide. Here, we report the death of two young Asian elephants after suffering from acute haemorrhagic disease due to EEHV-1A infection. We detected widespread distribution of EEHV-1A in various organs and tissues of the infected elephants. Enveloped viral particles accumulated within and around cytoplasmic electron-dense bodies in hepatic endothelial cells were detected. Attempts to isolate the virus on different cell cultures showed limited virus replication; however, late viral protein expression was detected in infected cells. We further showed that glycoprotein B (gB) of EEHV-1A possesses a conserved cleavage site Arg-X-Lys/Arg-Arg that is targeted by the cellular protease furin, similar to other members of the *Herpesviridae*. We have determined the complete 180 kb genome sequence of EEHV-1A isolated from the liver by next-generation sequencing and de novo assembly. As virus isolation in vitro has been unsuccessful and limited information is available regarding the function of viral proteins, we have attempted to take the initial steps in the development of suitable cell culture system and virus characterization. In addition, the complete genome sequence of an EEHV-1A in Europe will facilitate future studies on the epidemiology and diagnosis of EEHV infection in elephants.

## 1. Introduction

Asian elephants (*Elephas maximus*) are an endangered animal species. Existence of the species is further threatened by the emergence of a lethal herpesvirus, elephant endotheliotropic herpesvirus (EEHV). Infection with this virus can cause a devastating haemorrhagic disease, mostly in young Asian elephants between one and eight years of age, with up to 85% mortality. In older animals, the infection is predominantly inapparent [1,2]. The disease is characterized by sudden onset of illness, lethargy, edema, mild diarrhea, and sudden death with fatal haemorrhages in all visceral organs. Acute death of a young Asian elephant with haemorrhagic disease of unknown aetiology was first reported in 1988 [3]; retrospectively, the cause of the reported disease was identified as EEHV [1]. Since then, more than 100 confirmed cases of deaths of young elephants due to EEHVs have been reported in Asia, Europe, and North America [4,5]. EEHV infections have been reported mostly in Asian elephant calves in zoos. However, few deaths have also been documented in free-range Asian and African elephant calves [1]. Tracking elephant health in the wild is difficult, which may contribute to the low number of documented EEHV cases of wild elephants.

EEHVs are members of the family *Herpesviridae*, and are allocated to genus *Proboscivirus* in the subfamily *Betaherpesvirinae*. Seven distinct genotypes and five major subtypes of EEHVs have been identified: EEHV-1A and -1B, EEHV-2, EEHV-3A and -3B, EEHV-4A and -4B, EEHV-5A and -5B, EEHV-6 and EEHV-7A, and -7B [4,6]. Although infection of elephants has been reported with all genotypes of EEHVs, mortalities were mainly associated with EEHV-1A and -1B [4].

Diagnosis of the disease largely relies on clinical signs as well as the age of the elephant, detection of viral nucleic acids in blood samples or trunk washes using quantitative PCR (qPCR), post-mortem lesions, histopathology, and immunohistochemistry [7]. Despite decades of research, many aspects of disease pathogenesis, virus dissemination and transmission, predilection sites in host, sources of infection, and virus biology remain unclear. This may be partially attributable to the fact that the virus is not cultivable in vitro and that no small animal models are available. There is no vaccine or reliable therapeutic option available. Development of suitable cell culture systems and characterization of the virus remain the focus of current studies, with the hope to arrive at sustainable control measures in the future [8,9]. 

Here, we report on the death of two young Asian elephants, which occurred within a period of eight days after suffering from acute haemorrhagic disease in Tierpark Hagenbeck, Hamburg, Germany. Our molecular investigation revealed EEHV-1A as the cause of death. We performed pathological and virological investigations on infected tissues, including virus dissemination and viral load studies. We further performed transmission electron microscopy (TEM) of negatively stained sections of liver, tongue, and spleen tissues to visualize virus particles. To isolate the virus, we attempted virus propagation on different cell lines and assessed virus replication by qPCR, indirect immunofluorescence (indirect IF), and western blot (WB) assays. Taken together, we demonstrated the presence of EEHV particles in the infected tissues, showed limited viral replication in cell cultures, and provided evidence for the possibility of viral glycoprotein B (gB) cleavage similar to other members of the *Herpesviridae* family.

## 2. Materials and Methods 

Case history: The sudden death of a male Asian elephant “Kanja” (two years and five months of age) at Tierpark Hagenbeck was reported on 6 June 2018 after a short period of illness. “Kanja” was housed in the zoo along with nine other Asian elephants and showed colic-like clinical signs, depression, and anorexia before death. Two days later, the in-contact female elephant “Anjuli” (two years and 11 months old) showed an elevated body temperature without clinical signs. Immediately, before viral infection was diagnosed, Anjuli was treated with virostatic drugs (Famvir 500 mg (Famciclovir, Novartis Pharma GmbH, Vienna, Austria: 15 mg/kg TID (three times a day) orally for three days; she was then given tablets rectally for two days when she was sedated, as recommended by the the vet advisors of the EAZA (European Association of Zoos and Aquaria), Elephant Taxon Advisory group and previous reports [10]. The infected elephant developed clinical signs, failed to recover, and eventually died on 13 June 2018. In both cases, none of the elephants had a previous history of illness, but had not been previously tested for EEHVs. After the death of the two young elephants, as a precautionary measure, blood samples and trunk washes were collected from the other eight apparently healthy in-contact elephants and tested for EEHV-1A infection. 

Samples collection from elephants: Whole blood was collected from both elephants immediately before and after death. Complete necropsy was performed immediately after death and tissue samples were collected. All methods were carried out in accordance with the relevant guidelines and regulations according to the National Animal Protection Act (Behörde für Gesundheit und Verbraucherschutz, Fachbereich Veterinärwesen; approval number AFF012–EWG, 30 September 2009). Tissues and blood samples for virological diagnostics were collected and shipped to the Institut für Virologie, Freie Universität Berlin, in virus transport medium [serum-free MEM (Pan^TM^ Biotech, Aidenbach, Germany) with 100 U/mL penicillin (Panreac^TM^, AppliChem GmbH, Darmstadt, Germany) and 100 µg/mL streptomycin (Alfa Aesar^TM^, Thermo Fisher Scientific, Kandel, Germany) (1% P-S), 40 µg/mL gentamicin (Alfa Aesar^TM^, Thermo Fisher Scientific, Kandel, Germany), and 2.5 µg/mL amphotericin B (Biochrom GmbH, Berlin, Germany)] at 4 °C. Tissue samples for transmission electron microscopy (TEM) were collected in 10% phosphate-buffered formalin and re-fixed in Karnovsky’ fixative (7.5% glutaraldehyde and 3% paraformaldehyde; pH 7.4). Peripheral blood mononuclear cells (PBMC) were isolated from heparinised whole blood samples collected from infected elephant by Biocoll^®^ (Biochrom GmbH, Berlin, Germany) based density gradient centrifugation as described by the manufacturer. Briefly, whole blood samples were incubated for 30 min at room temperature (RT) for plasma rich PBMC separation from whole blood. Separated PBMC rich plasma was layered over Biocoll^®^ solution and centrifuged at 300 x g for 30 min. PBMC at the interphase were collected and washed twice in phosphate-buffered saline (PBS; 137 mM NaCl, 2.7 mM KCl, 10 mM Na_2_HPO_4_, and 1.8 mM KH_2_PO_4_). Isolated PBMC were used for co-cultivation culture and viral DNA isolation. 

DNA isolation and qPCR: DNA was isolated from PBMC (10^5^ cells), tissue samples (200 µg) (*viz*. mammary gland, muscle, bone marrow, adrenal gland, kidneys, spleen, pancreas, urinary bladder, liver, gall bladder, lymph nodes, blood vessels, lung, trunk, thyroid, temporal gland, cerebrum, cerebellum, stomach, small intestine, colon, uterus, tonsil, salivary gland, aorta, thymus, spinal cord, and heart), and infected cell cultures using innuPREP virus DNA/RNA kit^®^ (Analytik Jena™, Überlingen, Germany). DNA was extracted from whole blood using innuPREP blood DNA mini kit^®^ (Analytik Jena™, Überlingen, Germany). qPCR was performed using the StepOnePlus^TM^ real-time PCR system (Applied Biosystems, Foster City, CA, USA). Primers and probes specific to EEHV-1 were used as described previously and as seen in Table 1 [11,12]. The 20-µL reaction mixture contained 5 µL of extracted DNA sample, 10 µL of SeniFAST™ Probe Lo-ROX (2x) (Meridian Life Sciences, Inc. Memphis, TN, USA), 10 pmoli/µL of forward and reverse primer (0.9 µL of each), 10 pmoli/µL of probe (0.2 µL), and 3 µL of nuclease-free water. The thermal profile for cycling conditions was: hold for 2 min at 95 °C, 40 cycles of amplification (3 s at 95 °C and 30 s at 60 °C with data collection), and hold for 1 min at 60 °C for data collection. Standard curves were created using a synthetic 123-base pair length oligonucleotide of EEHV-1 terminase gene (GenBank Accession number: KC618527.1; Integrated DNA Technologies^®^, Coralville, Iowa) and quantified by measuring the absorbance at 260/280 nm using Nanodrop (NanoDrop Technologies, Thermo Fisher Scientific, Dresden, Germany) and an online application for calculating DNA copy numbers (http://www.sciencelauncher.com/mwcalc.html). Amplification efficiency (E > 90%) was calculated from the slope of the standard curve (with a correlation coefficient: R^2^ > 0.98) generated from 10-fold serial dilutions of the terminase gene oligonucleotide. EEHV-1 genome copies in the samples were compared with the generated standard curves. Positive DNA previously extracted from positive samples [12]) and negative (PBS) controls were used and included in every run. All samples were run in duplicates and samples were considered negative if the C_T_ value was > 39. Viral genome copies were then normalized to a standard curve generated with host specific oligonucleotide of elephant TNFα, as seen in Table 1 and as reported previously [8]. EEHV-1 DNA concentration was expressed as copies per million cells given that the eukaryotic cells of each diploid elephant have two copies of the TNFα gene. 

Cell culture: Crandell–Rees feline kidney (CrFK), Madin-Darby canine kidney II (MDCK II), African green monkey kidney (Vero), bovine dermal (BD), rabbit kidney 13 (RK-13), human embryonic kidney nuclear domain 10 deletion mutant [13] (293T ND10), and equine endothelial cells [14] (EC; kindly provided by Prof. Dr. Johanna Plendl, Freie Universität Berlin, Institut für Veterinär-Anatomie) were propagated in Dulbecco’s modified Eagle’s medium (DMEM) (Biochrom™, GmbH, Berlin, Germany), supplemented with 10% fetal bovine serum (FBS; Pan™ Biotech, Aidenbach, Germany) and 1% P-S. Equine dermal (ED) (CCLV-RIE 1222, Federal Research Institute for Animal Health, Germany) were grown in Iscove’s modified Dulbecco’s medium (IMDM; Pan™ Biotech, Aidenbach, Germany), supplemented with 20% FBS, 1 mM sodium pyruvate (Pan™), 1% nonessential amino acids (NEA; Biochrom™ GmbH, Berlin, Germany), and 1% P-S. Elephant fibroblast (ENL-2) (CCLV-RIE 856, Federal Research Institute for Animal Health, Greifswald, Germany) were grown in a 1:1 mixture of IMDM and Ham’s F12 (Biochrom™ GmbH, Berlin, Germany) with 20% FBS and 1% P-S. Human rectal tumour cells [15] (HrT-18G) were grown in DMEM supplemented with 10% FBS, 2 mM glutamine (Pan™ Biotech, Aidenbach, Germany), 1% NEA, 1 mM sodium pyruvate, and 1% P-S. Whole blood collected from the 54-year-old healthy elephant “Tanja” from Berlin Zoologischer Garten, which had tested negative for EEHV-1, was used for PBMC isolation using Biocoll^®^-based gradient centrifugation as described earlier. After washing in PBS, PBMC were re-suspended in RPMI 1640 medium (Pan™ Biotech, Aidenbach, Germany), supplemented with 10% FBS, 2 mM glutamine, 1% NEA, and 1% P-S. Blood was collected during the routine health checkup according to the National Animal Protection Act (Tierschutzgesetz; approval number D-AFF005–EWG, 18 March 1996).

Attempts of virus isolation in cell culture system: Tissue inoculums for virus isolation was prepared as described previously [16]. Briefly, tissue samples collected from the elephant “Kanja” (tongue, spleen and whole blood) were homogenized in the presence of cold PBS with 2% P-S and 2.5 µg/mL of amphotericin B, incubated on ice for 10 min, and frozen at −80 °C. Tissue homogenates clarified by centrifugation at 5000× *g* for 10 min were used for infecting different cells. Each cell type was grown in 24-well plates, and 200 µL of the clarified homogenate was added, incubated for 1 h at 37 °C, and 300 µL of the respective medium was added. Inoculated cultures were observed daily for the appearance of cytopathic effect (CPE) for seven days. When there was no CPE, inoculated cells were blindly passaged five consecutive times. Similarly, PBMC from infected elephants were either grown separately in culture medium or co-cultured with other cell lines, including PBMC from the healthy elephant.

Indirect immunofluorescence (IF) assay: Immunofluorescence staining was performed to detect herpesvirus antigen in cell culture as described previously [7,16]. ENL-2, CrFK, and PBMC were incubated with 200 µL of infected tongue tissue homogenate. After 96 h, cells were washed in PBS, fixed with 4% paraformaldehyde for 30 min, and permeabilized with 0.1% Triton X-100 for 10 min. Cells were blocked with 3% bovine serum albumin in PBS (Blocking buffer; VWR^TM^ Life Sciences, Dresden, Germany) for 30 min at RT, followed by incubation with primary rabbit anti-EEHV-1-gB antibodies [7] diluted 1:500 in blocking buffer at RT overnight. Secondary antibody, Alexa Fluor 488 goat anti-rabbit (Thermo Fisher Scientific, Dresden, Germany) diluted 1:500 in blocking buffer, was incubated for 1 hr at RT. Mock-infected cells were immunostained with primary and secondary antibodies at the same dilutions. Stained plates were examined and photos were taken using a Zeiss Axiovert.A1 fluorescent microscope, equipped with an Axiocam 503 camera. 

Western blotting: Cell lysates prepared from infected tongue tissue homogenate (from “Kanja”) were subjected to sodium dodecyl sulfate-polyacrylamide gel electrophoresis (SDS-PAGE) analysis. Briefly, 200 mg tissue samples were snap-frozen in liquid nitrogen, subjected to homogenization using hand-held tissue homogenizer and lysis using radioimmunoprecipitation assay buffer (50 mM Tris (pH 7.4), 0.25% Na-deoxycholate, 150 mM sodium chloride, 1 mM ethylene diamine tetra acetic acid; EDTA). Sample buffer (1 M Tris-HC1 (pH 6.8), 0.8% SDS, 0.4% glycerol, 0.15% β-mercaptoethanol, 0.004% bromophenol blue) was added to protein lysates. The samples were heated at 95 °C for 10 min and proteins were separated by 12% SDS-PAGE as described previously [17]. Expression of gB was detected with anti-gB antibodies, which either recognize the peptide sequence located between amino acids 259 and 274 (Ab7125) or between 427 and 441 (Ab7123) of EEHV-1 gB as derived from its sequence (GenBank accession NO. AF411189) [18]. Bound gB antibodies were detected with anti-rabbit IgG peroxidise conjugate. Reactive bands were visualized using enhanced chemiluminescence (ECL Prime, Amersham^TM^, GE Healthcare, United Kingdom). For non-reducing SDS-PAGE analyses, protein samples were prepared without β-mercaptoethanol as described previously [19].

Prediction of furin cleavage sites: Furin cleavage sites in EEHVs gB were predicted using the online tool ProP 1.0 Server (Technical University of Denmark, Lyngby, Denmark). To predict and compare the sites in gB of different EEHVs, amino acid sequences were retrieved from Uniprot database. It is predicted that the minimal cleavage site for furin is Arg-X-X-Arg or Arg-X-Lys/Arg-Arg [20]. 

lllumina library preparation and sequencing: For NGS library preparation, DNA was extracted from tissue samples [tongue “Anjuli” and liver “Kanja”] using the innuPREP Virus DNA/RNA Kit (AnalytiK Jena^TM^, Überlingen, Germany) as described above. Total DNA (5 µg) was diluted in 130 µL TE buffer and fragmented to a peak fragment size of 500 bp using the Covaris M220 focused-sonicator with appropriate settings. For size selection, the resulting DNA fragments (fragment size of 500–700 bp) were gel-purified after 1% agarose gel electrophoresis. The purified DNA was subsequently used to generate Illumina libraries using the NEBNext Ultra II Library Prep Kit for Illumina platforms (New England Biolabs, Ipswich, Massachusetts) according to the manufacturer’s instruction. To complete the adaptor sequences and to achieve a library yield > 500 ng and complete the adaptor sequences, 8 PCR cycles were performed at the end of the protocol. The index-amplified libraries were quantified using NEBNext Library Quant Kit for Illumina (New England Biolabs, Ipswich, Massachusetts) and a StepOnePlus^TM^ Instrument (Applied Biosystems, Foster City, CA, USA). Following quantification, samples were pooled to equimolar amounts to achieve a library concentration of 4 nM. The library pool was diluted further to load a final amount of 16 pM onto an Illumina MiSeq machine (Illumina Inc., Hayward, CA, USA) for DNA sequencing. 

Data analysis: NGS read data was used for genome assembly of the viral genomes. Reads were preprocessed using Trimmomatic [21] (version 0.36) for adapter and base quality trimming, only retaining reads of a minimal length of 100 bp after trimming. Reads were filtered by mapping against a pan genome sequence (BWA-MEM [22] version0.7.17) containing all available whole genome assemblies of EEHV; only read pairs with at least one read mapping the pan genome were used for final assembly. Quality filtered reads were assembled de novo using the SPAdes [23] assembler (version 3.13.0). Resulting contigs were further corrected using Pilon [24] (version 1.23) and scaffolded against reference genome (GenBank accession NO. KC462165.1) with Ragout [25] (version 2.1.1). In addition, reads mapping the pan genome (not quality trimmed) were mapped against the reference genome KC462165.1 using the mapping assembler MIRA [26] (version 4.9.6). Wherever possible, unmapped regions in the MIRA assembly caused by high sequence variation were filled with the SPAdes assembly to obtain a consensus genome. In a final step, this consensus was then used as reference for another mapping assembly using MIRA to generate the final sequence. Phylogenetic analyses were performed on nucleotide sequences obtained from whole genome sequencing. Nucleotide sequences of the terminase and vGPCR genes were used for the analyses. Reference sequences of same genes of EEHV-1 were retrieved from GenBank. Phylogenetic analysis was performed by maximum-likelihood method using MEGA 7.0.26 software. Branching was supported by bootstrapping with 1000 sets of data.

Transmission electron microscopy: Tissue samples of “Kanja’s” liver, tongue and spleen were converted from fixation by 10% formalin to fixation by Karnovsky solution (7.5% glutaraldehyde and 3% paraformaldehyde) as mentioned earlier, then washed in 0.1M cacodylate buffer (cacodylic acid sodium salt trihydrate; Roth; Karlsruhe, Germany) and subsequently fixed and contrasted for 4 hr in 1% osmium tetroxide (Roth; Karlsruhe, Germany). Samples were dehydrated in an ascending series of ethanol and in intermedium propylene oxide (1.2 epoxypropane; VWR^TM^ Life Sciences, Dresden, Germany), and afterwards embedded in a mixture of agar 100 (epoxy resin), dodecenylsuccinic anhydride (plasticizer), methylnadic anhydride (hardener), and DMP 30 (catalyst) (all: Agar Scientific Ltd.; Stansted, Essex, United Kingdom). Polymerization took place at 45 °C and 55 °C for 24 h. Semi- and ultrathin sections were cut at an ultra-microtome Reichert Ultracut S (Fa. Leica, Wetzlar, Germany). Semi-thin sections (0.5 µm) were stained with modified Richardson solution (Stain Technology 35:313–323, 1960) for 45 s on an electric hotplate adjusted to 80 °C. The semi-thin sections were checked by light microscopy Olympus CX 21 (Fa. Olympus; Stuttgart, Germany), and areas for ultra-thin sections were selected where the presence of the viruses in the cells was suspected. Ultrathin (80 nm) sections were mounted on Nickel-grids (Agar Scientific Ltd.; Stansted, Essex, United Kingdom) and examined with an electron microscope (Zeiss EM 109; Oberkochen, Germany). The digital photos were edited with Adobe Photoshop Program (Adobe Systems, Unterschleissheim, Germany). 

## 3. Results

### 3.1. Hemorrhagic Lesions in All Organs 

**Figure 1:**
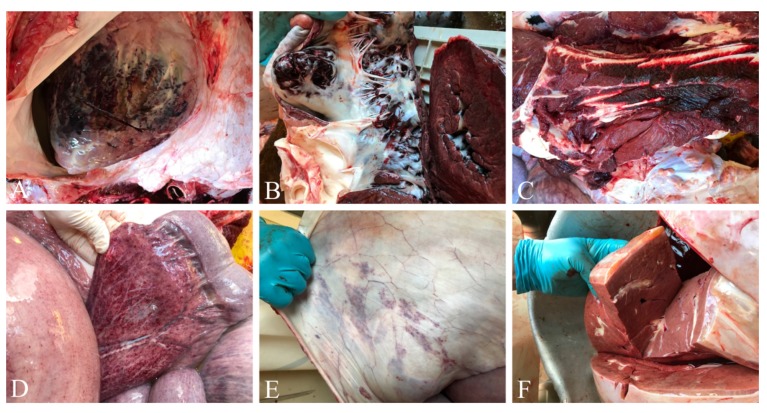
Necropsy of elephant endotheliotropic herpesvirus (EEHV-1A)-infected elephants. Post-mortem examination revealed severe petechial hemorrhages on the epicardial and endocardial surface of the heart with accumulations of sero-sanguinous fluid in the pericardium (**A**,**B**). Intermuscular hemorrhages in skeletal muscles (**C**), petechial hemorrhages in the mesentery (**D**) and thoracic wall (**E**), and peri-hepatic gelatinization of fat (**F**) were common findings.

At necropsy, gross examination revealed severe vascular lesions in all organs, as seen in Figure 1A–F. Prominent pathological changes were extensive haemorrhages in heart, skeletal muscles, small and large intestines, and lung. The heart showed severe and generalized endocardial and myocardial congestion, as seen in Figure 1A, B. Abdominal cavity revealed ascites with sero-sanguineous fluid accumulation (about five liters). Hemorrhages in skeletal muscles, congestion of mucosal surface of trunk, pharynx, larynx and lung, subcutaneous edema, and cyanosis of tongue were important findings, as seen in Figure 1C–F. In addition to pericardial effusion, diffuse petechial hemorrhages were observed on the serosal surfaces of stomach, intestine, mesentery, omentum, urinary bladder, and abdominal wall. Small and large intestine mucosal surfaces were edematous, hemorrhagic, and congested. Post-mortem lesions were very similar in both deceased elephants. 

### 3.2. Extensive Distribution of Virus in All Organs

DNA was extracted from all collected tissues and blood, and qPCR analysis was performed. Because the virus is endotheliotropic in nature and viremia is the common finding during infection, it was expected that all tissues would be positive for EEHV-1. However, we surmised that quantification of viral genome copies in each tissue might help in understanding predilection sites for virus replication. Thirty-eight samples were collected from the infected elephants “Kanja” and “Anjuli” and qPCR analysis was performed. All tissues were positive for EEHV-1 and most of tissues had virus genome copies in the range of 10^6^–10^8^ copies per million cells, as seen in Table 2. Viral DNA load was very high in bone marrow, heart, liver, urinary bladder, trunk, axillar lymph node (LN), tongue, and muscle with a range of 3.17 × 10^8^ − 5.11 × 10^7^ copies per million cells. All remaining samples had virus genome copies between 4 × 10^7^−1.5 × 10^6^ per million cells. Inguinal LN, gall bladder, cerebellum, and prescapular LN had the lowest range of viral genome copies (5.25 × 10^3^ − 8.72 × 10^4^ copies per million cells). Viral DNA load in tissues from “Kanja” was almost similar and within a comparable range without much difference. 

### 3.3. Trials of Virus Isolation in Cell Culture Revealed Limited Virus Replication

**Figure 2:**
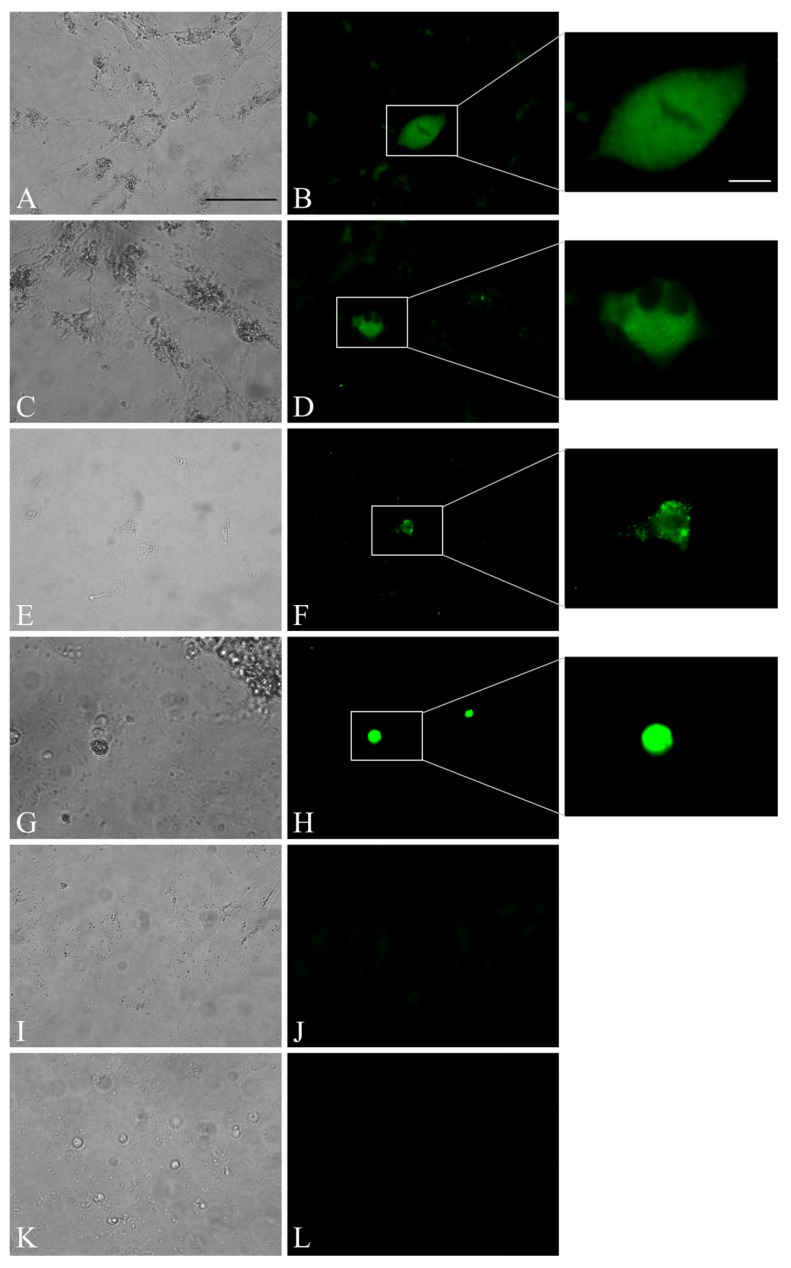
Indirect IF staining of EEHV-1 gB 96 h post inoculation. Indirect IF revealed expression of gB of EEHV-1 in ENL-2, CrFK, PBMC inoculated with tongue homogenate. (**A**,**C**,**E**,**G**) Bright green IF-staining of cytoplasm of the EEHV-1 infected ENL-2 (**A**,**C**), CrFK (**E**), and PBMC (**G**). (**B**,**D**,**F**,**H**) Corresponding bright-field images for **A**, **C**, **E**, and **G**. (**I**,**K**) Uninfected negative control staining shows no IF signals for ENL-2 (**I**) and healthy PBMC (**K**). (**J**,**L**) Corresponding bright-field images for **I** and **K**. Scale bar is 50 µm. Inserts: magnification of corresponding positive immunofluorescence signals. Insert scale bar is 10 µm.

Tissue (tongue, spleen, and whole blood; “Kanja”) homogenates and infected PBMC from “Kanja” and “Anjuli” were inoculated into 10 different cell lines and the PBMC collected from the healthy elephant “Tanja”. Upon inoculation, cells were observed daily for the presence of CPE. As no CPE was observed, cells were blindly passaged continuously five times. qPCR analysis and indirect IF were performed to assess the infectivity and replication of the virus. qPCR analysis revealed limited replication of EEHV-1 in ENL-2 (elephant) cells, endothelial (equine) cells, CrFK (feline) cells, and 293T (ND10-knocked down, human) cells until passage 3, as seen in Table 3 and Table 4. Interestingly, elephant fibroblast cells (ENL-2) supported virus replication and maintained viral nucleic acids until passage 4 when co-cultured with infected PBMC (from both “Kanja” and “Anjuli”). Indirect IF results confirmed expression of EEHV-1 gB in cell cultures. The positive immunofluorescence signals in ENL-2 cells, CrFK cells, and elephant PBMCs at 96 h post-infection revealed that these cells were infected by the virus and further supported virus replication and late protein expression to some extent, as seen in Figure 2A–J. Although the virus infected these cells, no clear CPE was observed at any passage, and the cells did not support virus replication beyond passage 4. On the other hand, tissue samples inoculated on MDCK II, Vero, ED, RK-13, BD, HrT-18G cells did not support EEHV-1 replication after the first passage as all inoculated samples tested negative for EEHV-1 DNA in qPCR. 

### 3.4. Expression and Cleavage of gB 

**Figure 3:**
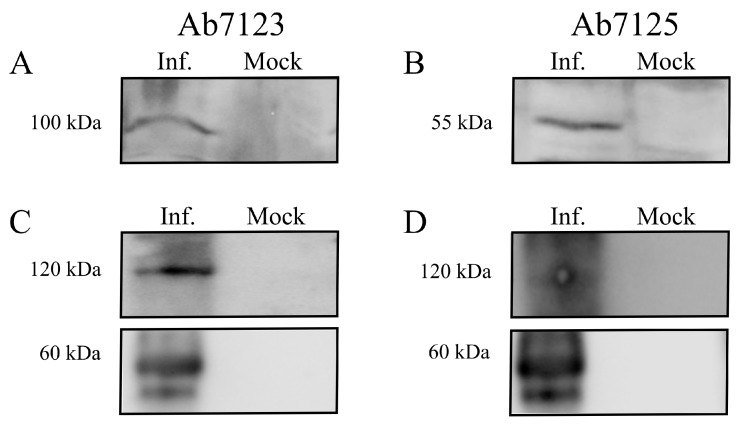
(**A**,**B**) Western blot analysis was performed under reducing conditions using antibodies raised against EEHV-1 gB (Ab7123 and Ab7125). Ab7123 (**A**) reacted with a band of 100 kDa (uncleaved gB) while Ab7125 (**B**) detected cleaved gB with a size of 55 kDa. (**C**,**D**) Western blot under non-reducing conditions, in which both Ab7123 and Ab7125 reacted with proteins of 120 kDa and 60 kDa size. Cropped western blot images given in **C** and **D** were from the same gel and probed with Ab7123 and Ab7125, respectively. Inf.—infected tissue, Mock—healthy tissue.

**Figure 4:**
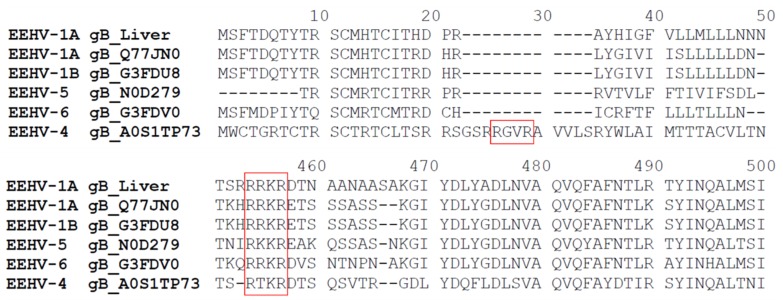
Furin cleavage site prediction among other gBs of EEHVs. Red boxes mark the predicted furin cleavage sites. EEHV-1A gB_Liver: gB sequence obtained from the current study.

SDS-PAGE was performed for protein lysates obtained from frozen tongue tissues of EEHV-1 infected elephant “Kanja”. A signal of the band corresponding to the predicted gB protein size of 100 kDa was observed using Ab7123 antibodies (recognizing the peptide sequence located between amino acids (aa) 427 and 441 of gB), as seen in Figure 3A. A second band of 55 kDa was observed when Ab7125 antibodies (recognizing the peptide sequence located between aa 259 and 274 of gB) were used, as seen in Figure 3B. The predicted size of the EEHV-1 gB gene is 2553 bp, which encodes a 97 kDa protein (850 aa). However, when we performed WB from infected tissue homogenates using two different antibodies raised against different regions of gB (aa259–274 and aa427–441), we observed two bands with different sizes. We performed a prediction (ProP v.1.0b ProPeptide Cleavage Site Prediction) for the presence of possible protease cleavage sites, which revealed the presence of a furin recognition motif (^433^RRKR^436^) recognized by cellular propeptide protease and can lead to the cleavage of gB into two subunits of 50.5 and 46.5 kDa, as seen in Figure 4. Further analysis of available gB sequences of other EEHVs from GenBank revealed the presence of one or two furin cleavage sites in a similar fashion: one motif in EEHV-1B (^429^RRKR^432^), EEHV-5 (^416^RKKR^420^), and EEHV-6 (^429^RRKR^432^) and two motifs in EEHV-4 (^26^RGVR^29^ and ^447^RTKR^450^). The Ab7125 antibodies were raised against the peptide sequence ^259^EPSTKFKVYKDYERLQ^274^, whereas Ab7123 antibodies were raised against the peptide sequence ^427^ANVTSRRRKRDANTA^441^ covering the furin cleavage site in gB of EEHV-1 (Uniprot: Q8JTJ0). Hence, Ab7125 antibodies detected the cleaved *N*-terminal part of gB while Ab7123 antibodies detected uncleaved gB, as evidenced by the 55 kDa and 100 kDa bands, respectively. Further, non-denaturing native SDS-PAGE yielded gB-specific bands of around 60 kDa and 120 kDa with both Ab7123, as seen in Figure 3C, and Ab7125, as seen in Figure 3D. 

### 3.5. Transmission Electron Microscopy (TEM) Revealed Intranuclear and Intracytoplasmic Viral Particles

**Figure 5:**
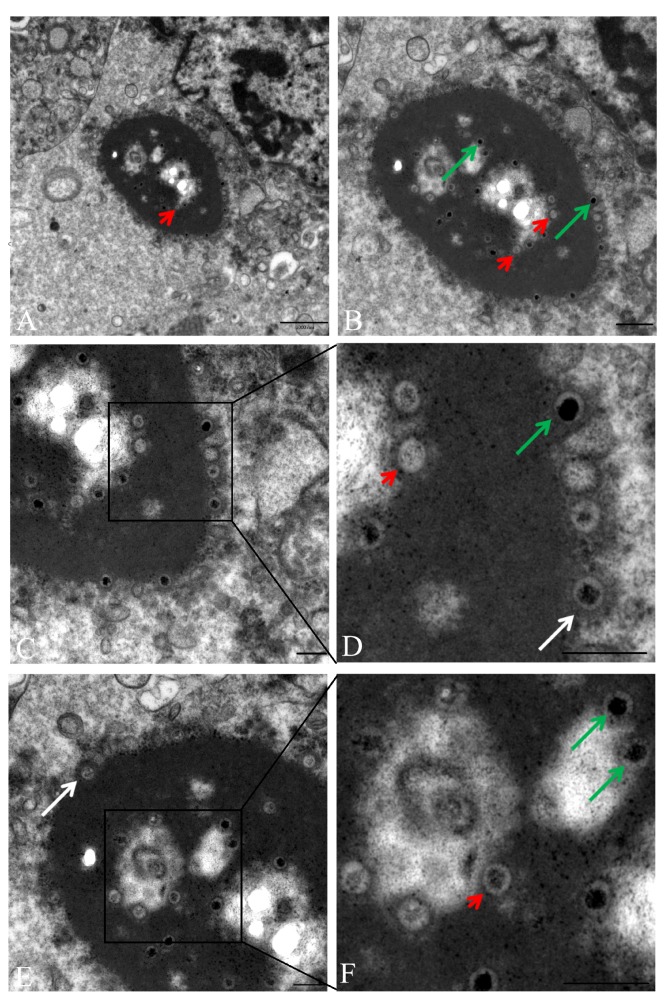
Transmission electron microscopy. (**A**) Hepatic endothelial cells contained cytoplasmic and intranuclear viral particles (scale bar 1000 nm). (**B**) Viral particles are accumulated in paranuclear cytoplasmic electron-dense bodies (scale bar 500 nm). (**C** and **E**) Electron-dense cytoplasmic matrix contained nucleocapsids with electron dense cores and capsids with electron lucent cores. Tegument formation was visualized around the nucleocapsids (scale bar 250 nm). **D** and **F** are magnification of **C** and **E**, respectively (scale bar 250 nm). Green arrow—nucleocapsids with electron dense core; white arrow—nucleocapsids and capsids, surrounded by a tegument; Red arrowhead—capsid with electro-lucent cores.

For the detection of EEHV-1 virions, liver, tongue, and spleen tissues were ultrastructurally examined. Within hepatic endothelial cells, cytoplasmic and intranuclear virus particles were detected, as seen in Figure 5A. Intranuclear virus particles (empty and DNA-containing nucleocapsids) were found at the marginal zone of the endothelial nucleus. In addition, a large number of virus particles was accumulated within and around an electron-dense cytoplasmic matrix, which was located in close proximity to the cell nucleus, as seen in Figure 5B. The electron-dense cytoplasmic matrix contained nucleocapsids with electron-dense and electron-lucent cores. The capsids measured approximately 80 nm and the cores 60 nm in diameter. At the periphery of the electron-dense cytoplasmic body, tegument formation was visualized around the nucleocapsids and the empty capsids, as seen in Figure 5C–F. Virus particles with tegument had a diameter of approximately 120 nm. Tongue and spleen tissues also showed ultra-structural lesions along with virus like particles in the cytoplasm of endothelial cells. 

### 3.6. Whole Genome Sequencing of EEHV-1A

The datasets for the liver and tongue samples comprised a total of ~12.5 and ~8.4 million paired-end reads with ~6.9 and ~6.0 million pairs passing quality filtering. Removing read pairs not mapping the pan-genome produced a set of ~43,000 read pairs for liver but only ~9000 read pairs for tongue. The reads of the tongue sample were not sufficient to produce a high-quality assembly of the virus genome and was excluded from further analysis. For the liver sample “Kanja”, the reference-based scaffolding of SPAdes contigs using Ragout created a scaffold of length ~169.5 kbp containing the two longest contigs with ~161.4 kbp and ~8.2 kbp (average coverage of ~18.8 and ~15). The initial mapping assembly with MIRA against the ~180 kbp reference genome KC462165.1 contained 426 ambiguous and 7422 uncovered bases. After using the SPAdes de novo assembly to fill gaps in the MIRA consensus and to run MIRA again with this augmented consensus, the numbers were reduced to 295 and 4919, respectively. The complete viral DNA genome of EEHV-1A has been determined and annotated from liver sample “Kanja” as described earlier and has 98.52% identity with the available EEHV-1A sequences from the GenBank. The obtained sequence of EEHV-1A genome was deposited in GenBank under accession number MN067515. 

**Figure 6:**
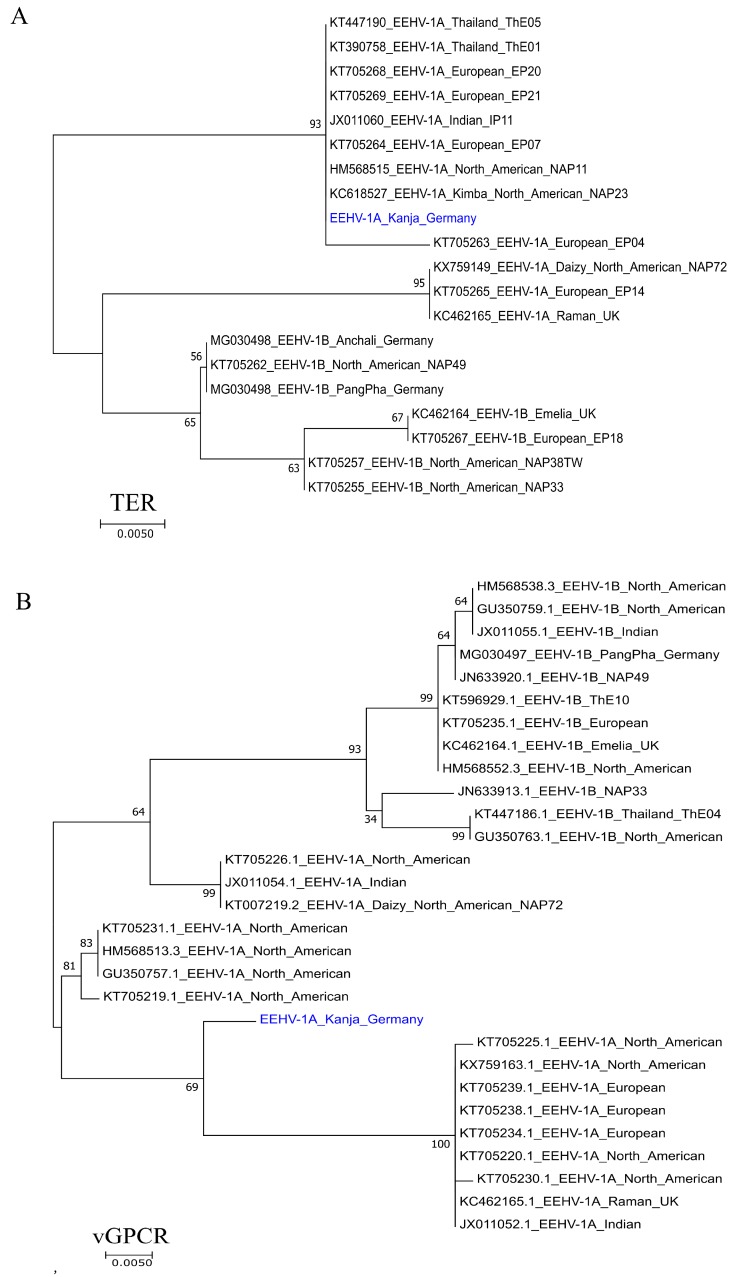
Phylogenetic tree of EEHV-1 DNA. Maximum-likelihood trees are shown for the terminase (**A**) and vGPCR (**B**) genes of EEHV-1. EEHV-1A “Kanja” sequence from current study was indicated in blue. Reference sequences obtained from GenBank are represented by their accession numbers. Bootstrap values above 50% are shown. The scale bar indicates nucleotide substitution per site.

Genetic relatedness of the current EEHV-1A was further determined by phylogenetic analysis of the terminase and vGPCR genes. At the nucleotide level, terminase gene sequence obtained from “Kanja” was clustered with several EEHV-1A isolates from North America, Europe, and Asia, as seen in Figure 6A. On the other hand, the vGPCR gene sequence, although clustering with other EEHV-1A isolates, formed a separate clade. 

## 4. Discussion

Elephants are facing a serious infection threat since EEHVs became one of the main causes of death of young elephants in captivity in the last three decades. The viruses, which were described to cause skin nodules and local infections in the 1970s, have caused severe fatal hemorrhagic disease outbreaks since the late 1980s [1,3,27,28,29]. Today, EEHVs are considered ubiquitous in Asian elephant populations in captivity and in free range throughout the world [6,9,30]. Like other herpesviruses, EEHVs seem to have successfully co-evolved with their hosts and established a well-balanced adaptation. However, incidence of peracute disease in young elephants remains peculiar as this contradicts the herpesvirus survival strategy exclusively adapted for long-term persistence in the host [31].

In the present study, we identified that EEHV-1A was the cause of the death of two young elephants consecutively in Tierpark, Hagenbeck. In each case, the elephant showed clinical signs just before death for a brief period of time, did not respond to treatment, and eventually died. Lesions of haemorrhagic nature were observed in most of the visceral organs and tissues including heart, liver, lung, intestine, mesentery, tongue, and pharynx. Similar findings of acute onset of illness and sudden death due to haemorrhagic disease in EEHV-1A-infected juvenile elephants has been described previously as the virus infects epithelial and endothelial cells [1,4,7,30]. We detected EEHV-1A DNA in various organs and tissues of the infected elephants, as was reported earlier [31]. Widespread distribution of viral nucleic acid might be attributed to the extensive virus replication, at least in the endothelial lining, of all tissues. However, the portal of virus entry, target sites, receptors involved, and the primary site of virus replication remain unclear.

Electron microscopy revealed non-enveloped viral particles of about 120 nm in diameter accumulated within and associated to paranuclear cytoplasmic electron-dense bodies in hepatic endothelial cells. Aggregation of fully formed enveloped virus particles at the proximity within cytoplasmic dense bodies have been reported twice [3,32]. In all cases, cytoplasmic dense bodies were observed only in the liver. Capillary endothelial cells of the liver showing basophilic intracytoplasmic viral inclusion bodies in histopathology were reported previously [33]. This finding can be correlated with the paranuclear electron-dense bodies in the cytoplasm observed by electron microscope. The reason of cytoplasmic dense bodies’ formation specifically in the endothelium of the liver and its role in virus replication is not yet understood. It can be surmised that the liver supports virus replication better than other organs, as viral nucleic acid load is very high in liver tissues in comparison to other tissues. Virus particles in different steps of virus replication were also observed in endothelial cells of tongue and spleen tissues, which confirmed that virus replication always occurs in endothelial cells of different tissues and contributes to high viral load in different organs. Similarly, the presence of virus particles in endothelial cells of spleen [2] and heart [1,29] has been previously shown.

It was previously reported that EEHV-1 could not be isolated on cell culture system [3]. Here, we attempted to isolate the virus on different cell cultures, and we observed limited virus replication for few passages. We used different cell lines originating from different species including elephant, human, equine, bovine, canine, rabbit, feline, and monkey. Among the different cells used, only elephant fibroblast cells (ENL-2) showed limited virus replication for up to four passages (28 days) when inoculated with infected tongue tissue homogenate and co-cultured with infected elephant PBMC. This finding lead to the assumption that elephant cells are a potential platform for virus replication in vitro. However, after the fourth passage, no viral DNA was detected in infected ENL-2 cells, which again limits the use of these cells. Equine endothelial cells and 293T (with ND10 knockdown) human cells showed persistence of viral genomes up to three passages. As reported earlier [3], virus replication was not supported in other cell lines tested. One can argue that the reduction of DNA copies in cells was mainly due to a dilution effect. However, this was not the case with all cell lines. The DNA disappeared completely from some cell cultures after the first passage but continued in others until the fourth passage, as seen in Table 3 and Table 4. Moreover, the viral genome copies fluctuated between low and high with ENL-2 cells when co-cultured with infected PBMC. We propose that (i) EEHV-1 preferentially replicates in cells derived from its natural host, i.e., elephant; (ii) endothelial cells may support virus replication longer than other cells from different hosts; and (iii) deletion of host restriction factors (such as ND10) that block virus replication may help in supporting EEHV-1 replication. Another possible reason for the overall less efficient virus replication in cell culture is the lack of suitable tissue complexity required for virus replication as provided in the natural host.

In addition to qPCR, indirect IF demonstrated the expression of viral proteins (gB) in infected cells (ENL-2, CrFK and PBMC) at 96 hrs post infection, which was not shown before. Although expression of viral protein (gB) was observed in both ENL-2 and CrFK cells, only ENL-2 cells supported virus replication as shown in qPCR results. Herpesvirus gBs are highly conserved, homologous among other members, and essential for virus replication [34,35,36,37]. Across the members of the *Herpesviridae*, gB possesses a conserved cleavage site Arg-X-Lys/Arg-Arg targeted by the cellular protease furin. Here, we show that EEHV-1 gB has also a furin cleavage site like other members [17]. gB of EEHV-1A has a conserved furin cleavage motif ^433^RRKR^436^, which cleaves gB in the middle. In western blotting, antibodies raised against two different peptides of gB detected both cleaved and uncleaved forms of gB. This may point to the importance of gB cleavage for the protein to be functionalized. Furthermore, we conducted a prediction analysis for gB sequence of other EEHVs available online, which revealed that all available EEHVs (EEHV-1A, 1B, 4, 5, and 6) also have at least one furin cleavage site. As no information is available regarding the function of any protein of EEHV-1, this study makes the initial step in describing the similarity of gB among different EEHVs in terms of furin cleavage, which was highly conserved across the family of herpesviruses. We therefore assume that gB plays an important role in virus entry and egress from target cells, as it is described for other herpesviruses [38,39,40].

We determined the whole 180 kb genome sequence of EEHV-1A in liver tissue by next-generation sequencing and de novo assembly. EEHV-1A genome analysis of our current study confirms the relatedness of the virus to other reported whole genome sequences of EEHV-1A. The phylogenetic analysis of terminase and vGPCR genes showed clustering of EEHV-1A sequences (of the current study) with other isolates of EEHV_1A of other countries in North America, Europe, and Asia. The complete EEHV-1A genome sequence will provide a basis for better understanding of epidemiology, host-pathogen interaction, and evolution of EEHV-1 virulence.

## 5. Conclusions

Taken together, we have examined two fatal cases of EEHV-1A in young Asian elephants in captivity. We have studied the pathology of the virus in both elephant calves, demonstrated viral particles in liver endothelium, and showed widespread viral distribution in host tissues. Our attempt to isolate EEHV-1A in established cell lines showed interesting results as cells derived from elephants supported virus replication for four passages. Additionally, we found that gB of EEHV-1 possesses a conserved cleavage site targeted by cellular furin protease. This pattern is also known for other members of the *Herpesviridae* and this cleavage is required to generate a functional glycoprotein. Finally, the whole EEHV-1A DNA genome was sequenced from one of the cases: “Kanja”. The findings and facts of this study will be helpful for the development of suitable cell culture system and further characterization of EEHVs with respect to developing prophylactic strategies and implementing control measures in future.

## Figures and Tables

**Table 1:** Primers, probes, and oligonucleotides used for qPCR.

**EEHV-1_Ter_For**	actgcaaaygcattcttaaaagat
**EEHV-1_Ter_Rev**	agaatgggattrgctaagaagct
**EEHV-1_Ter_Probe**	tcaacgaggagatattaggcaccaccaaca
**EEHV-1_Ter_Oligo**	cattgacactggaatctgttagaatgggattggctaagaagctcgtgttggtggtgcctaatatctcctcgttgaacgaatcttttaagaatgcgtttgcagtttttttgatattcaaattaa
**Ele_TNFα_For**	cccatctacctgggaggagtct
**Ele_TNFα_Rev**	tcgagatagtcaggcagattgatc
**Ele_TNFα_Probe**	ccagctagagaagggt
**Ele_TNFα_Oligo**	tgaggccaagccctggtatgagcccatctacctgggaggagtcttccagctagagaagggtgatcgactcagcgctgagatcaatctgcctgactatctcgactttgccgagtctgggcaggtca

**Table 2:** qPCR of the terminase gene of EEHV-1 in collected tissue samples. Normalized viral genome copies in various tissue samples collected from the infected elephant “Anjuli” at necropsy were given. Mean values of viral genome copies were calculated from two replicates for each sample.

Tissues	Normalized Viral Genome Copies	Tissues	Normalized Viral Genome Copies
Bone marrow	3.17 × 10^8^	Lung	1.19 × 10^7^
Heart	2.25 × 10^8^	Temporal gland	1.15 × 10^7^
Liver	1.65 × 10^8^	Colon	1.00 × 10^6^
Urinary bladder	9.49 × 10^7^	Adrenal gland (left)	7.00 × 10^5^
Trunk	6.79 × 10^7^	Uterus	5.62 × 10^5^
Axillar LN	6.71 × 10^7^	Mesenteric LN	5.55 × 10^5^
Tongue	5.36 × 10^7^	Adrenal gland (right)	5.25 × 10^5^
Muscle	5.11 × 10^7^	Thymus	3.81 × 10^5^
PBMC	4.04 × 10^7^	Thyroid	3.46 × 10^5^
Cervical LN	3.98 × 10^7^	Cerebrum	3.44 × 10^5^
Tonsils	3.04 × 10^7^	Small intestine	3.17 × 10^5^
Mammary gland	2.97 × 10^7^	Kidney	2.68 × 10^5^
Stomach	2.89 × 10^7^	Pancreas	2.37 × 10^5^
Trunk mucosa	2.88 × 10^7^	Spinal cord	2.50 × 10^5^
Spleen	2.87 × 10^7^	Salivary gland	1.53 × 10^5^
Mandibular LN	2.34 × 10^7^	Prescapular LN	8.72 × 10^4^
Aorta	1.69 × 10^7^	Cerebellum	7.79 × 10^4^
Blood vessel	1.46 × 10^7^	Gall bladder	7.05 × 10^4^
Blood	1.37 × 10^7^	Inguinal LN	5.25 × 10^3^

**Table 3:** qPCR of the terminase gene of EEHV-1 in infected ENL-2 and elephant PBMC co-culture. qPCR was performed on DNA extracted from cell cultures (cells and supernatant separately) after inoculation with tissue samples from infected elephants from passage 1 to passage 5. Mean values of viral genome copies for each sample were calculated from two replicates. Cell: cell pellet; Sup: supernatant; -: negative.

Tissue	Cell Line	Pass 1		Pass 2		Pass 3		Pass 4		Pass 5	
		Cell	Sup	Cell	Sup	Cell	Sup	Cell	Sup	Cell	Sup
Tongue “Kanja”	ENL-2	12775	136614	2018	5128	-	212	-	-	-	-
											
PBMC “Kanja”	ENL-2	1944	4233	110	301	-	-	-	56	-	-
PBMC “Anjuli”	ENL-2	36011	27753	-	604	91	1279	-	538	-	-
	PBMC	19873	15322	976	392	-	-	-	-	-	-

**Table 4:** qPCR of the terminase gene of EEHV-1 in infected cultures. qPCR was performed on DNA extracted from cell cultures (cells and supernatant separately) after inoculation with tissue samples from infected elephants from passage 1 to passage 5 and C_T_ values were given. Cell: cell pellet; Sup: supernatant; -: negative.

Tissue	Cell Line	Pass 1		Pass 2		Pass 3		Pass 4		Pass 5	
		Cell	Sup	Cell	Sup	Cell	Sup	Cell	Sup	Cell	Sup
**Tongue** **“Kanja”**	CrFK	30	25	37	32	-	-	-	-	-	-
	MDCK II	32	25	-	37	-	-	-	-	-	-
	Vero	29	23	-	34	-	-	-	-	-	-
	BD	32	28	-	32	-	-	-	-	-	-
	RK-13	31	25	-	37	-	-	-	-	-	-
	293T	31	26	37	33	-	38	-	-	-	-
	ED	30	24	-	32	-	-	-	-	-	-
	EC	29	24	36	31	-	37	-	-	-	-
**PBMC** **“Kanja”**	CrFK	33	31	-	37	-	-	-	-	-	-
	MDCK II	34	31	-	38	-	-	-	-	-	-
	Vero	31	31	-	-	-	-	-	-	-	-
	BD	35	36	-	37	-	-	-	-	-	-
	RK-13	34	31	37	-	-	-	-	-	-	-
	293T	34	32	-	-	-	-	-	-	-	-
	ED	36	34	37	36	-	-	-	-	-	-
	EC	32	31	35	35	-	37	-	-	-	-
**Blood** **“Kanja”**	CrFK	38	29	-	-	-	-	-	-	-	-
	MDCK II	-	30	-	-	-	-	-	-	-	-
	Vero	38	29	-	-	-	-	-	-	-	-
	BD	38	35	-	38	-	-	-	-	-	-
	RK-13	-	30	-	-	-	-	-	-	-	-
	293T	38	30	-	38	-	-	-	-	-	-
	ED	-	29	-	-	-	-	-	-	-	-
	EC	33	29	-	35	-	-	-	-	-	-
**Spleen** **“Kanja”**	CRFK	32	25	-	33	-	-	-	-	-	-
	MDCK II	34	26	-	35	-	-	-	-	-	-
	Vero	33	26	-	36	-	-	-	-	-	-
	BD	33	28	-	33	-	-	-	-	-	-
	RK-13	31	25	-	36	-	-	-	-	-	-
	293T	32	26	-	34	-	-	-	-	-	-
	NBL-6	34	25	-	34	-	-	-	-	-	-
	EC	37	25	-	36	-	-	-	-	-	-
**PBMC** **“Anjuli”**	HrT-18G	29	29	38	38	-	-	-	-	-	-
